# Case Report: Avoiding misdiagnosis in amyloidosis—navigating transthyretin genopositivity and monoclonal gammopathy in a patient with advanced heart failure and spinal stenosis

**DOI:** 10.3389/fcvm.2024.1479676

**Published:** 2024-12-16

**Authors:** Xia Wu, Denis Toskic, Ping Zhou, Stephanie Scalia, Xun Ma, Parva Bhatt, Teresa Fogaren, Monika Pilichowska, Knarik Arkun, Jainith Patel, Ron I. Riesenburger, Daniel P. Larson, Raymond L. Comenzo

**Affiliations:** ^1^ Division of Hematology-Oncology, Department of Medicine, Tufts Medical Center, Boston, MA, United States; ^2^ The John Davis Myeloma and Amyloid Program in the Cancer Center, Tufts Medical Center, Boston, MA, United States; ^3^ Department of Pathology, Tufts Medical Center, Boston, MA, United States; ^4^ Department of Neurosurgery, Tufts Medical Center, Boston, MA, United States; ^5^ Department of Laboratory Medicine and Pathology, Mayo Clinic, Rochester, MN, United States

**Keywords:** ATTR amyloidosis, AL amyloidosis, cardiac amyloidosis, ligamentum flavum (LF), differential diagnosis

## Abstract

**Background:**

A 63-year-old Black woman presented with progressive exertional dyspnea and chronic lower back pain. The course and findings in her case are instructive.

**Case report:**

Family history was notable for cardiac deaths. An echocardiogram demonstrated ventricular wall thickening with diastolic dysfunction. The patient’s N-terminal pro b-type natriuretic peptide level was 1,691 pg/ml with a troponin I level of 0.36 ng/ml. Transthyretin (TTR) sequencing detected a heterozygous V122I variant. The patient’s free *κ* light chain level in serum was 664 mg/L with a ratio of 16.5. Bone marrow analysis showed 20%–30% κ-restricted plasma cells with amyloid deposits. A technetium-99m sodium pyrophosphate scan was performed and was negative. Magnetic resonance imaging of the total spine showed ligamentum flavum (LF) thickening at L4–5, causing severe spinal stenosis. In both the abdominal fat and the LF, liquid chromatography-coupled tandem mass spectrometry confirmed κ-type immunoglobulin light chain (AL) amyloidosis; the quantitative estimate of amyloid content in the LF was 5%. She was diagnosed with AL amyloidosis with Mayo Stage IIIA cardiac and soft tissue involvement, enrolled in the Aquarius trial (NCT05250973) in Cohort 2, and received daratumumab, cyclophosphamide, bortezomib, and dexamethasone. She achieved a partial hematological response with a cardiac response and is now pain-free and fully functional.

**Conclusion:**

In patients with amyloidosis who have both monoclonal gammopathy and a TTR variant, it is imperative to discern the tissue type of the amyloid to deduce the correct diagnosis. ATTR and AL amyloidosis can both cause spinal stenosis with minimal degenerative changes. The LF tissue must be stained for amyloids and, if present, typing must be performed.

## Introduction

Amyloidosis is a family of diseases driven by misfolded or misassembled precursor proteins that deposit in organs and tissues and cause symptoms. Immunoglobulin light chain (AL) amyloidosis and transthyretin (ATTR) amyloidosis are two common types of systemic amyloidosis and both often involve the heart. With novel agents and new combinations of therapies, the prognosis of both AL and ATTR amyloidosis has improved significantly (1, 2). However, late-stage cardiac involvement and delays in diagnosis are correlated with poor prognosis (3). Therefore, early and accurate diagnosis and subtyping of amyloidosis are critical to ensure appropriate treatment to improve outcomes.

Diagnosis of AL or ATTR amyloidosis can be challenging, especially with cardiac involvement when patients could have similar clinical presentations and echocardiogram findings. In patients with evidence of both monoclonal gammopathy and a TTR mutation, correct subtyping is even more challenging. Given monoclonal gammopathy, clinicians may infer that AL amyloidosis is the diagnosis; however, “Occam's Razor” can be misleading, particularly in Black individuals, among whom monoclonal gammopathies occur at twice the rate observed in white individuals, while mutated TTR genes are present in 3.9% of Black individuals, especially the p.Val122Ile (V122I) mutation (4). Therefore, in the presence of monoclonal gammopathy, tissue typing is mandatory to verify the amyloid composition to achieve the correct diagnosis (5). Here we present an instructive case in point.

## Case description

**Figure 1:**
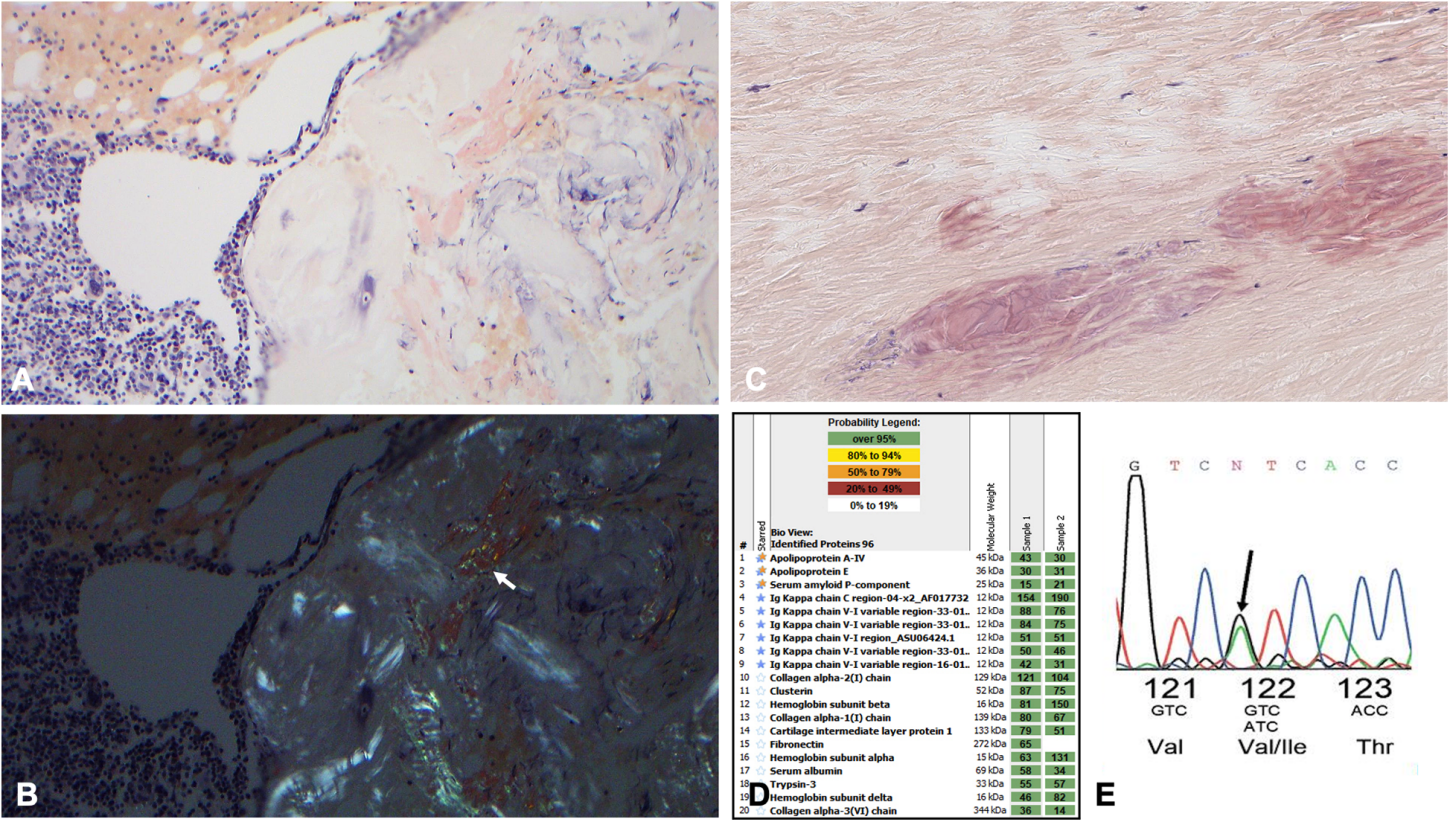
**(A)** 200× Congo red staining highlighting focal amyloid deposition in the patient's bone marrow specimen. **(B)** Corresponding polarized light images of the bone marrow specimen. The arrow is pointing to the apple green birefringence. **(C)** Congo red special staining highlights Congophilic deposits within the ligamentum flavum fibers at 200× magnification; the deposits are a dirty pink salmon color with delicate cracks, along with apple green polarization (not shown), consistent with amyloids. **(D)** The proteomic profile of the ligamentum flavum specimen. The columns labeled “sample” represent two separate analyses from laser micro-dissected Congo red-positive deposits in this case. The proteins highlighted with double stars represent universal amyloid proteins. The proteins highlighted with blue stars represent amyloid-specific markers detected in this case. The numbers in the green boxes correlate with the total number of MS/MS spectra detected of the particular protein in each row. **(E)** The Chromas TTR DNA sequence indicating the substitution of isoleucine (lle) for valine (Val) at position 122.

A 63-year-old Black woman from Cameroon presented with progressive fatigue and exertional dyspnea. She reported worsening dyspnea upon exertion for the previous 3 months, particularly during stair climbing. She denied having a cough, chest pain, diarrhea, abdominal pain, dysuria, fever, or weight changes. Her medical history was otherwise notable for worsening chronic lower back pain that was radiating down to her left leg and had been refractory to physical therapy and opioid analgesics. Her family history was notable for cardiac deaths, with her father dying at 59 and her sister in her teens, though details were unclear. Upon presentation, she was afebrile; her blood pressure was 136/84 mmHg, her heart rate was 97 beats per minute, and her oxygen saturation was 99% on room air. Physical examination showed regular heart rate and rhythm with no murmurs and clear lung sounds with no edema, cutaneous lesions, or macroglossia. A transthoracic echocardiogram demonstrated left ventricular wall thickening, interventricular septal thickness at end-diastole (IVSd) of 1.6 cm, ejection fraction of 45%, grade III diastolic dysfunction, and a severely dilated left atrium (Supplementary Figures S1C,D); strain imaging demonstrated an apical sparing pattern (Supplementary Figure S1E), which warranted a work-up for amyloid cardiomyopathy. Laboratory studies revealed that her N-terminal pro b-type natriuretic peptide (NT-proBNP) level was elevated to 1,691 pg/ml (normal range, 0–125) and her troponin I level to 0.36 ng/ml (<0.03). Her clinical picture raised the concern for cardiac amyloidosis; therefore, the patient was screened for monoclonal proteins. Both serum and urine immunofixation electrophoresis (IFE) showed free κ light chains without heavy chains. Serum protein electrophoresis (SPEP) did not show an M spike. Serum protein studies showed an elevated free κ light chain level at 664 mg/L (3.3–19.4) with a *κ*: *λ* ratio of 16.5 (0.5–2.0). A clonal plasma cell population of 20%–30% *κ* restricted CD138-positive cells was detected in the bone marrow, which contained translocation (11; 14), and marrow plasma cell staining was positive for cyclin D1 by immunohistochemistry. Congo red staining and examination in polarized light revealed amyloids in the marrow (Figures 1A,B) and abdominal fat and typing by liquid chromatography-coupled tandem mass spectrometry (LC/MS) confirmed κ-type AL. The sequence of the monoclonal amyloidogenic light chains was further identified to be IGKV1-33 (GenBank accession number: PP112602).

**Figure 2:**
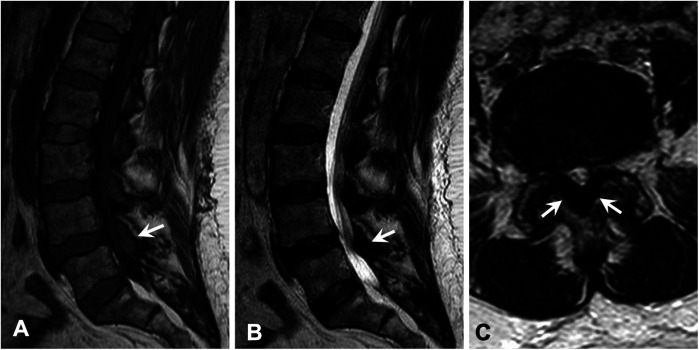
Magnetic resonance imaging of the lumbar spine demonstrated severe thickening of the ligamentum flavum at the level of L4–5 (arrow) with associated abnormal T1 hyperintensity, which caused severe narrowing of the spinal canal and bilateral neural foramina. Similar lesions were also seen at the levels of L1–4 to a lesser extent. **(A)** Sagittal T1-weighted image. **(B)** Sagittal T2-weighted image. **(C)** Axial T2-weighted image.

**Figure 3:**
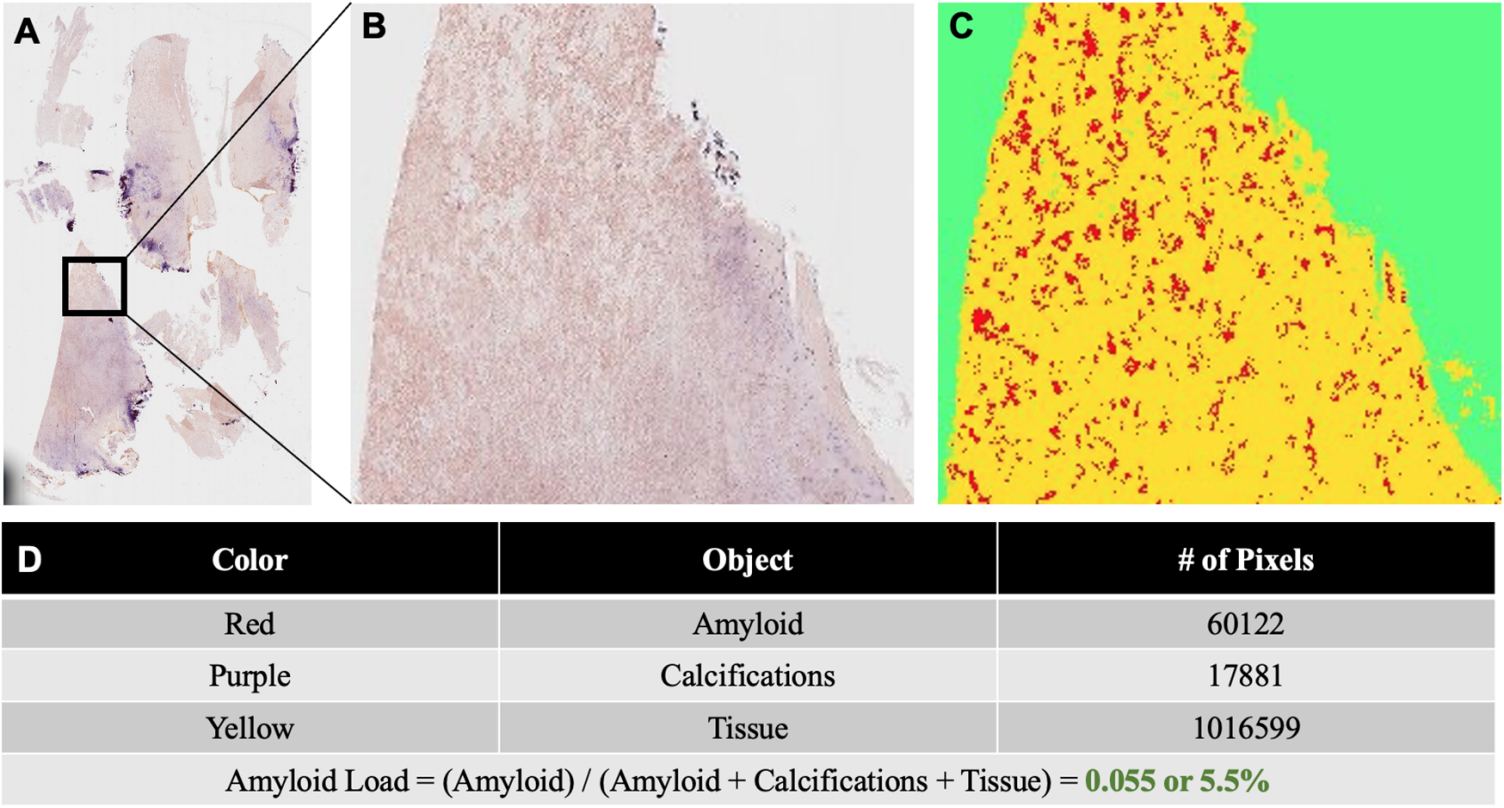
**(A)** Congo red stained slide of ligamentum flavum tissue resected at L4–5. The tissue was scanned under non-polarized light and digitized using a VENTANA DP 200 slide scanner (Roche Diagnostics, Rotkreuz, Switzerland) at 20× magnification and one focus layer. **(B)** Higher magnification of the Congo red stained ligamentum flavum specimen. **(C)** The results image generated for the quantification of amyloids and other tissue elements using the Trainable Weka Segmentation model. **(D)** Table displaying the total number of pixels corresponding to amyloids, calcifications, and tissue in the results image. Amyloid load was calculated as the total number of amyloid pixels divided by the sum of all pixels corresponding to amyloids, calcifications, and tissue.

As for the lower back pain, magnetic resonance imaging (MRI) of the lumbar spine showed severe thickening of the ligamentum flavum (LF) at the level of L4–5 and severe narrowing of the spinal canal and bilateral neural foramina (Figures 2A–C). Given the sciatica symptoms refractory to physical therapy and pain medications, the patient underwent an L4–5 laminectomy and onlay arthrodesis, with subsequent relief of her back pain. The removed thickened LF was sent for biopsy and showed positive Congo red staining; LC/MS confirmed κ-type AL (Figures 1C,D) and the calculated amyloid load was 5.5% (Figure 3). The amyloid load was defined as the percentage of LF tissue occupied by amyloids and was quantified using a previously validated machine-learning model developed using Trainable Weka Segmentation (Fiji Version 2.1.0/1.53c) (6). The entire specimen displayed in Figure 3A was utilized for amyloid quantification.

**Figure 4:**
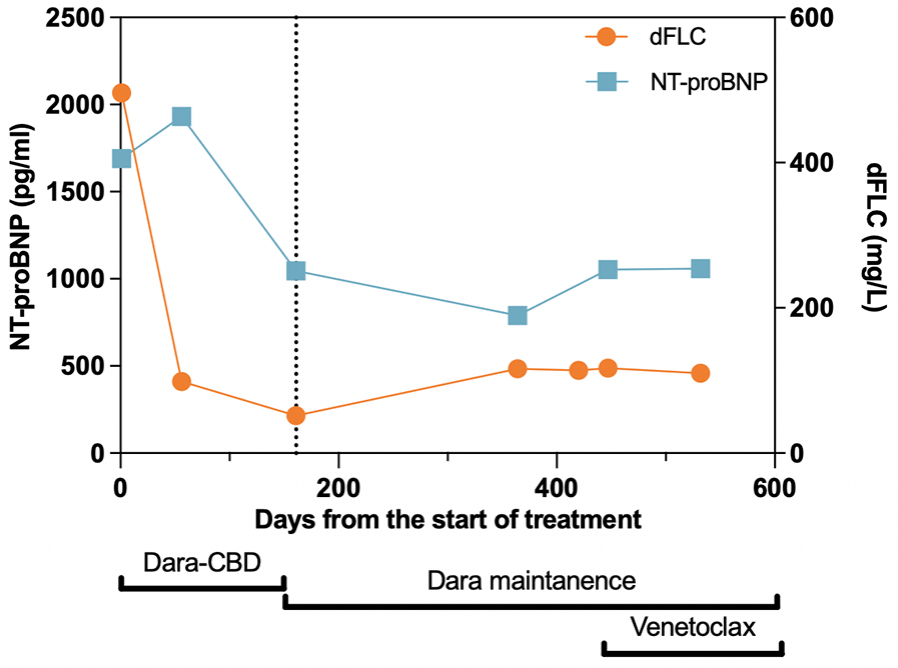
Treatment timeline with corresponding cardiac and hematological responses. The vertical dotted line represents the 6-month follow-up time point, at which the patient’s hematological and cardiac responses were assessed. At this time, the patient achieved partial hematological and cardiac responses, which were maintained throughout the follow-up period. Dara, daratumumab; CBD, cyclophosphamide, bortezomib, and dexamethasone; dFLC, difference between involved and uninvolved serum free light chains; NT-proBNP, N-terminal of prohormone brain natriuretic peptide.

Given the evidence of κ-type AL proteins in the soft tissue forming amyloids, the patient was suspected to have primary AL amyloidosis, with both cardiac involvement and soft tissue involvement. However, ATTR cardiac amyloidosis could not be ruled out without a further work-up given the patient's race and family history of cardiac death. To better identify the driver of cardiac disease, a 1 h technetium-99m (Tc-99m) sodium pyrophosphate (PYP) scan with single photon emission computed tomography (SPECT) was performed and showed grade 0 uptake on planar imaging with a heart-to-contralateral lung ratio of 1.1, which was not consistent with ATTR cardiac amyloidosis (Supplementary Figures S1A,B). We also performed TTR gene sequencing, detecting a heterozygous V122I TTR variant (Figure 1E). Therefore, she was diagnosed with AL amyloidosis with Mayo Stage IIIA cardiac and soft tissue involvement, enrolled in the Aquarius trial (NCT05250973), and received daratumumab, cyclophosphamide, bortezomib, and dexamethasone (Dara-CBD) (7). In addition, the patient also received torsemide and spironolactone for volume and electrolyte management. At the 6-month follow-up, she achieved a partial hematological response following six cycles of Dara-CBD, along with a partial cardiac response (8, 9). At the 18-month follow-up, the patient maintained partial hematological and cardiac responses. Her symptoms of back pain and exertional dyspnea had notably alleviated and she was fully functional (Figure 4).

## Discussion

Of the cardiac amyloidosis subtypes, AL and wild-type ATTR (ATTRwt) are relatively common, with hereditary ATTR (ATTRm) being less common (10, 11). Differentiating ATTRwt and ATTRm from the AL subtype can be challenging due to their similar patterns of organ involvement; both can present as restrictive cardiomyopathy, carpel tunnel syndrome, autonomic dysfunction, and spinal stenosis. Furthermore, both monoclonal gammopathies and ATTRm occur more frequently in African Americans (12–14). When a patient has evidence of both monoclonal gammopathy and a TTR mutation, it is even more challenging to identify which is the driving cause of symptoms. In a cohort analysis of 197 patients with ATTR amyloidosis, 42% had monoclonal gammopathy with a *κ* predominance (15).

The diagnosis of amyloidosis is demonstrated by evidence of amyloid deposition either histologically or via highly specific diagnostic imaging studies. In addition, certain imaging studies provide valuable insights into AL amyloidosis. For example, speckle-tracking echocardiography can detect apical sparing, also known as the “cherry on top” pattern, which is an informative feature of cardiac amyloidosis (16). To determine the subtype of amyloidosis, whether AL, ATTR, or another subtype, further studies are warranted in addition to history, physical exam, and other regular work-ups. For instance, SPEP, serum and urine IFE, and a free light chain assay are used to search for evidence of monoclonal proteins; TTR gene sequencing can identify a TTR gene mutation, and Tc-99m PYP and Tc-99m 3,3-diphosphono-1,2-propanodicarboxylic acid (DPD) scans can assist in diagnosing ATTR cardiac amyloidosis. In addition, cardiac magnetic resonance (CMR) imaging plays a crucial role in assisting the diagnoses of AL and ATTR amyloidosis by demonstrating differences in right ventricular size, myocardial strain, and patterns of late gadolinium enhancement (17–20). Moreover, recent studies have highlighted the promising role of CMR imaging in predicting prognosis and monitoring therapeutic responses in patients with AL cardiac amyloidosis (21, 22). Furthermore, LC/MS is the gold standard for amyloid subtyping (23).

Notably, the guidelines have clear recommendations regarding in which order the above diagnostic studies should be performed to diagnose AL, ATTR, or another cardiac amyloidosis appropriately (24). With clinical suspicion of cardiac amyloidosis, the first step is to screen for monoclonal gammopathy with SPEP, serum and urine IFE, and a free light chain assay; if evidence of monoclonal proteins is detected, the next step is a tissue biopsy with Congo red staining and possibly LC/MS to identify the type of amyloidosis; if monoclonal gammopathy is not found, then nuclear scintigraphy can be performed if available with a positive result being diagnostic for ATTR amyloidosis. If this is the case, ATTR genetic testing should also be performed to differentiate between ATTRwt and ATTRm; if the nuclear scintigraphy result is indeterminate, a cardiac biopsy should be considered and, if the result is negative, cardiac amyloidosis is less likely (25, 26). Recently, concerns have been expressed regarding the inappropriate use of Tc-99m PYP scans without completing a monoclonal protein evaluation as they could result in frequent false positive results in cases of AL amyloidosis, which subsequently hinders the real-world value of diagnosing ATTR using a Tc-99m PYP scan (27, 28). Therefore, following the diagnostic steps recommended by the guidelines is of great importance.

In our patient, we applied the same diagnostic algorithm as above but also considered her unique situation to avoid misdiagnosis. With the initial clinical symptoms and findings indicating cardiac and soft tissue amyloidosis, monoclonal protein studies were performed and a subsequent tissue biopsy with LC/MS showed systemic κ-type AL amyloidosis. We could have prematurely concluded that the patient had cardiac amyloidosis driven by amyloidogenic light chains. Nonetheless, taking her race and cardiac family history into account, the patient could have had distinct amyloid subtypes involving separate anatomic sites (ATTR with transthyretin amyloids in the heart), or even a combination of AL and ATTR amyloids in one site (the heart). Such diagnoses are rare yet have been reported in the literature (4, 29). To avoid a misdiagnosis leading to inappropriate treatment, Tc-99m PYP SPECT was performed, and with a grade 0 on planar imaging and a heart-to-contralateral lung ratio of 1.1, the cardiac amyloidosis was considered to be caused by AL instead of ATTR. Therefore, our case is instructive because it serves as a valuable supplement to the existing diagnostic algorithms in specific situations. It highlights the importance of incorporating an individualized approach, even when following established diagnostic algorithms, to achieve an accurate diagnosis of the type of amyloidosis and prevent potential mistreatment.

Notably, our patient also has a heterozygous V122I TTR variant, predisposing her to ATTRm amyloidosis. While our patient's current amyloidosis is light chain-driven, it is important to understand the possibility that two amyloid types may be present in one individual (4). In our patient, the V122I TTR variant may predispose her to develop hereditary ATTR cardiac amyloidosis in the future; however, there are currently no established guidelines for surveillance in such cases. Sidiqi et al. analyzed 1,094 patients with LC/MS-confirmed amyloids and identified nine (0.82%) patients who had two amyloid types. All nine patients had ATTR amyloids and six had AL amyloids (30). In such a case, a repeat Tc-99m-PYP scan and a repeat biopsy with amyloid typing should be considered to further evaluate the possible development of ATTR amyloidosis.

Studies have shown that amyloid deposits are commonly found in the LF of patients with spinal stenosis (31), with ATTR subtypes accounting for nearly 80% (32). In rare cases, patients can have both ATTR and AL amyloid deposits in the LF (33). In our case, the LF tissue had an amyloid load of 5.5%, a finding consistent with prior reports (34). Patients with ATTRwt amyloids in their LF have been shown to have less severe disc degeneration compared to patients with lumbar stenosis without amyloids. This suggests that amyloids may play a separate role in LF thickening compared to the degeneration of intervertebral discs (35). While the exact mechanisms by which AL and ATTR amyloids contribute to the thickening of the LF are relatively unknown, a positive association between amyloid load and the thickness of the LF has been established (34). Therefore, in patients with systemic amyloidosis who present with lower back pain, it is reasonable to consider potential amyloid deposits in the LF that cause LF thickening and spinal stenosis, and a histopathological work-up of the LF might assist a further diagnosis.

## Conclusion

In patients with suspected cardiac amyloidosis, screening for monoclonal gammopathy is required prior to further consideration of Tc-99m-PYP scans. However, in patients with cardiac amyloidosis who present with both monoclonal gammopathies and a TTR variant, it is imperative to ascertain the tissue type of the amyloids and to employ diagnostic modalities such as Tc-99m-PYP scans to deduce the correct diagnosis. In elderly patients who may have both monoclonal gammopathies and wild-type ATTR amyloidosis, the same guidance applies. Meanwhile, ATTR and AL amyloids can both cause spinal stenosis with minimal degenerative changes shown in spinal MRI. LF tissue must be stained for amyloids and, if present, typing must be performed.

## Data Availability

The original contributions presented in the study are included in the article/Supplementary Material, further inquiries can be directed to the corresponding author.
