# Supplementary material for: Case Report: Avoiding misdiagnosis in amyloidosis—navigating transthyretin genopositivity and monoclonal gammopathy in a patient with advanced heart failure and spinal stenosis
Source: Front Cardiovasc Med. 2024 Dec 16;11:1479676. doi: 10.3389/fcvm.2024.1479676 (PMC11683122; doi:10.3389/fcvm.2024.1479676)
Supplement: Supplementary file 1 [file Image1.pdf]

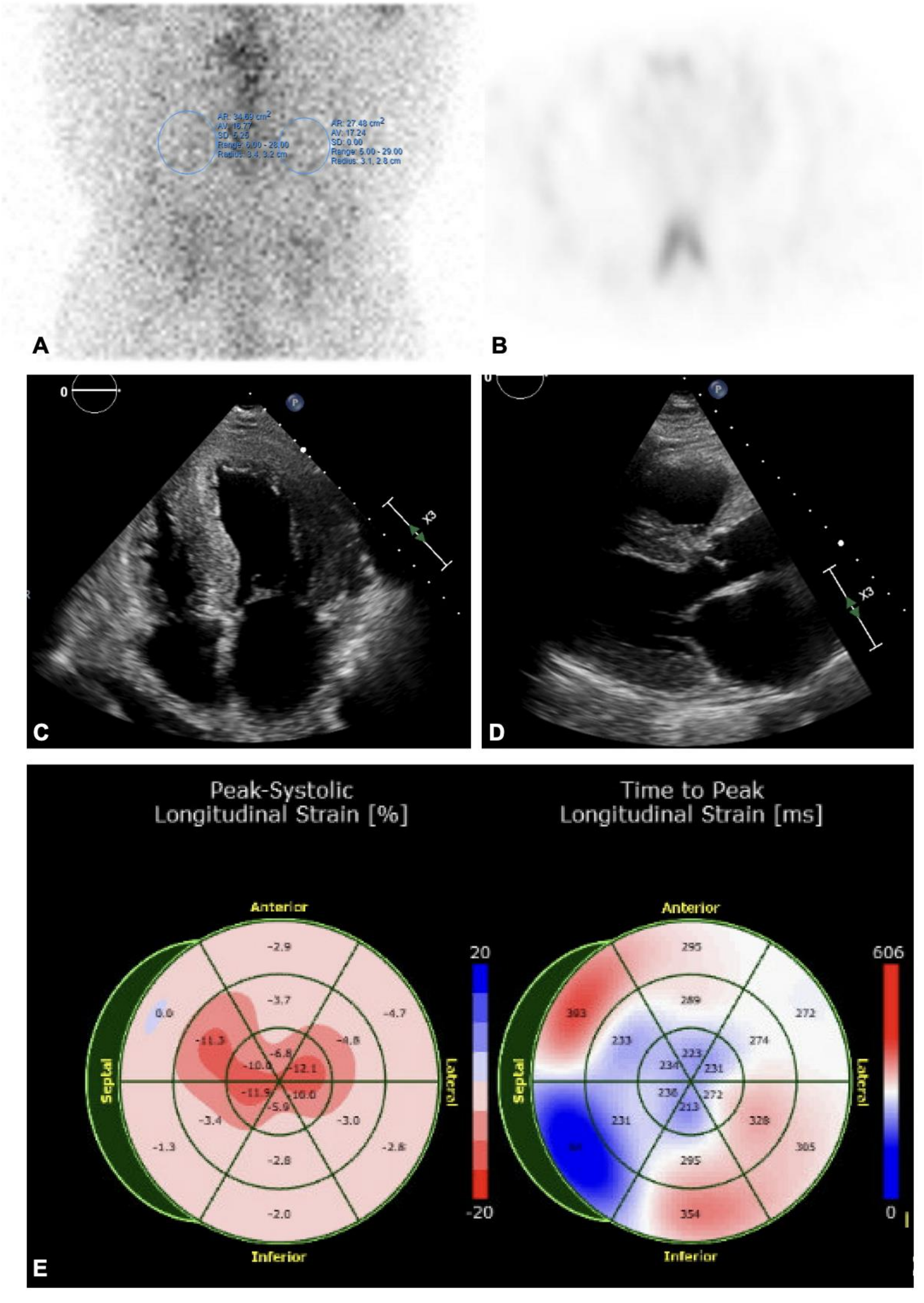
A
B
C
D
E
Peak-Systolic
Longitudinal Strain [%]
Time to Peak
Longitudinal Strain [ms]
Anterior
Septal
Lateral
Inferior
20
-20
606
0

Supplementary Figure 1

A-B: Technetium-99 m sodium pyrophosphate scan, with planar imaging (A) and single photon emission computed tomography (B) showing grade 0 uptake, with the heart-to-contralateral lung ratio 1.1, indicating not consistent with ATTR cardiac amyloidosis.

C-D: Echocardiography, with four chamber view (C) and parasternal long axis view (D) showing concentric left ventricular wall thickening and increased interventricular septal thickness.

E: Echocardiography strain imaging study showing the apical sparing pattern of longitudinal strain.
